# Consistent signatures of selection from genomic analysis of pairs of temporal and spatial *Plasmodium falciparum* populations from The Gambia

**DOI:** 10.1038/s41598-018-28017-5

**Published:** 2018-06-26

**Authors:** Alfred Amambua-Ngwa, David Jeffries, Roberto Amato, Archibald Worwui, Mane Karim, Sukai Ceesay, Haddy Nyang, Davis Nwakanma, Joseph Okebe, Dominic Kwiatkowski, David J. Conway, Umberto D’Alessandro

**Affiliations:** 1 Medical Research Council Unit The Gambia at LSHTM, Banjul, The Gambia; 2 London School of Hygiene and Tropical Medicine (LSHTM), London, UK; 3 Wellcome Trust Sanger Institute, Hinxton, UK

## Abstract

Genome sequences of 247 *Plasmodium falciparum* isolates collected in The Gambia in 2008 and 2014 were analysed to identify changes possibly related to the scale-up of antimalarial interventions that occurred during this period. Overall, there were 15 regions across the genomes with signatures of positive selection. Five of these were sweeps around known drug resistance and antigenic loci. Signatures at antigenic loci such as thrombospodin related adhesive protein (*Pftrap*) were most frequent in eastern Gambia, where parasite prevalence and transmission remain high. There was a strong temporal differentiation at a non-synonymous SNP in a cysteine desulfarase (*Pfnfs*) involved in iron-sulphur complex biogenesis. During the 7-year period, the frequency of the lysine variant at codon 65 (*Pfnfs*-Q65K) increased by 22% (10% to 32%) in the Greater Banjul area. Between 2014 and 2015, the frequency of this variant increased by 6% (20% to 26%) in eastern Gambia. IC_50_ for lumefantrine was significantly higher in *Pfnfs*-65K isolates. This is probably the first evidence of directional selection on *Pfnfs* or linked loci by lumefantrine. Given the declining malaria transmission, the consequent loss of population immunity, and sustained drug pressure, it is important to monitor Gambian *P*. *falciparum* populations for further signs of adaptation.

## Introduction

The success of malaria interventions such as artemisinin-based combination therapies (ACTs), seasonal malaria chemoprevention, long lasting insecticidal nets (LLIN) and indoor residual spraying (IRS) in reducing malaria burden has raised hopes for malaria elimination^1,2^. However, this enthusiasm is threatened by the emergence of artemisinin resistance (artR) in South East Asia (SEA)^3^, resulting into coordinated effort to limit its spread beyond SEA and detect any emergence in sub-Saharan Africa (sSA)^4^. Detailed surveillance for spatial and temporal trends in the frequencies of mutations associated with resistance to artemisinin and partner drugs is essential, particularly for the non-synonymous single nucleotide polymorphisms (SNP) in the Kelch propeller and BTB-POZ domains (KPBD) of the *P*. *falciparum* gene (*Pfk*1*3*, Pf3D7_1343700) that have been associated with delayed parasite clearance; the artemisinin resistance phenotype^5^. The prevalence of *Pfk*13 artR polymorphisms is high in SEA but low in sSA^6,7^. However, there are increasing reports that suggest reduced susceptibility of *P*. *falciparum* to ACTs beyond SEA, including cases of complete treatment failure in Africa^8–10^. These artemisinin-tolerant parasites may have been selected by the increased use of ACTs in the last decade. Such selection would leave genetic markers of adaptation in the *P*. *falciparum* population.

Population genetics approaches to identify markers of local adaptation often include comparison of genetic variation between spatially separated populations. In sSA, this has been demonstrated in pairwise comparisons of *P*. *falciparum* genomes between The Gambia and Guinea, two sites within Ghana, and across eight locations spanning ~1000 Km in Mauritania^11–13^. These studies assessed the allele frequency spectra and patterns of linkage disequilibrium, allowing for the identification of signatures of differentiation and positive selection, which could be due to local adaptation. Isolates from these sites were mostly collected over a single transmission season and do not provide information on the stability of genomic structure of the populations over a longer period, during which changes may occur, including coverage of control interventions. Temporal analysis has helped in understanding the evolution of drug resistance loci^14–16^. In addition, candidate markers for artemisinin resistance in *P*. *falciparum* isolates from SEA were identified by examining SNPs with allele frequency shifts similar to the *Pfk1*3-C580Y^17^ resistance marker. Presently, there is no reference marker for artR such as the *Pfk13*-C580Y for sSA parasites, so this approach may not be feasible. However, by comparing the genomes of isolates before and after the introduction of ACTs, genomic changes and signatures of sustained antimalarial drug pressure and decline in disease burden can be identified in settings such as The Gambia.

Between 2005 and 2015, the incidence of malaria in the Gambia dropped by over 75% following the widespread use of artemether-lumefantrine (AL) and high coverage of vector control interventions, namely LLINs and IRS^18,19^. Malaria transmission in the country has become increasingly heterogeneous, with prevalence of infection between ~5% and >40% in the west and eastern regions respectively^20^. AL was introduced as first-line treatment in 2008, replacing Sulfadoxine-Pyrimethamine (SP), which remains in use for intermittent preventive treatment during pregnancy and for seasonal malaria chemoprevention (SMC), combined with amodiaquine (since 2014)^21^. Chloroquine and other antimalarial monotherapy remains available and poorly regulated, in the private and informal sector (personal correspondence)^22^.

We recently reported an increasing proportion of *P*. *falciparum* isolates with *ex vivo* tolerance to lumefantrine in western Gambia^23^. Resistance to SP and other quinolines remain high^23^, with whole genome scans of the 2008 population showing strong signatures of selection around resistance markers of these drugs^14,24,25^. Building on this evidence, this study compared these earlier detected signatures to those of current populations. We detected sustained selection in genomic regions and new loci under differential selection that could have been selected for by drug pressure and other interventions.

## Results

### Temporal increase in linkage disequilibrium (LD), low complexity and spatial substructure between recent populations

**Figure 1:**
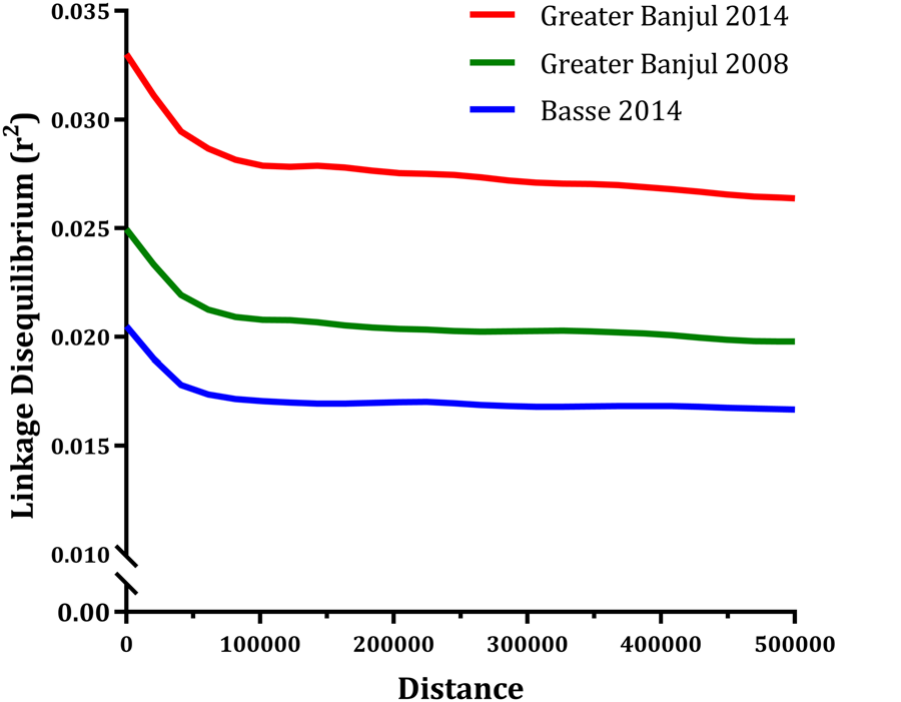
The decay of linkage disequilibrium (r^2^) with physical distance (kilobases) for coding single nucleotide polymorphisms in three Gambian *P*. *falciparum* populations. Each line is a smoothed fit of the pairwise linkage disequilibrium against distance per population; Greater Banjul 2008, 2014 and Basse 2014.

Linkage disequilibrium between 16,629 coding SNPs was low in all three populations, decaying rapidly below an r^2^ of 0.05 within 5000 base pairs (Fig. 1). Long range LD was evident mainly for the 2008 and 2014 populations from Greater Banjul (GBJ), (Supplementary Fig. 1). The extent of LD decay correlated with parasite prevalence; being highest for the 2014 population from Basse followed by the 2008 and then 2014 GBJ populations. Median infection complexity determined by the inverse measure of outcrossing (Fws) was low for all populations despite a narrow reduction in Fws values for the GBJ 2014 samples (median Fws from 0.91 to 0.78) compared to 2008. Median Fws for Basse was 0.83, and not significantly different from the GBJ 2008 population. Isolates from the three populations did not form significantly distinct clusters by principal component analysis (PCA), based on 10,862 low LD SNPs. However, dimension 1 and 2 of the PCA analysis showed low-level separation of the recent 2014 spatial populations from GBJ and Basse, though with significant overlap of the geographic clusters (Supplementary Fig. 2). There was a low genome wide fixation index (0.014, SD 0.019), which confirms a lack of significant structure between the spatial population pair (GBJ and Basse from 2014). The spatial fixation index was higher than that observed between the temporal population pair of 2008 and 2014 from GBJ, which was 0.009, with a standard deviation of 0.0166.

### Consistent selective sweeps around drug resistance and antigenic loci in all populations

Regions that have undergone positive selection would show reduced heterozygosity and extended haplotypes^26^. Based on the standardised integrated haplotype score (|iHS|), we identified 1066SNPs within 11 regions of hard sweeps on chromosomes 1, 4, 6, 7, 8, 9, 11, 13 and 14 (Supplementary Fig. 3). There were seven consistent sweeps across all populations and these included the strongest known *P*. *falciparum* sweep that spans the chloroquine resistance transport (*Pfcrt*) on chromosome 7. The only two SNPs that reached the significance threshold for |iHS| in the recent 2014 population from GBJ were on coding regions of conserved proteins located 60kbp from *Pfcrt* (PF3D7_0710200, PF3D7_071350). The sweep on chromosome 11 spanning *Pfama1* gene was only significant in the GBJ 2008 population, while that spanning the *Pfmsp1* and *Pftrap* genes on chromosomes 9 and 13, respectively were the strongest in the 2014 Basse population. Other antigenic loci within sweep signatures included the SURFINs (1.1, 4.2 and 8.2) and merozoite surface proteins 7–like gene cluster on chromosome 13. |iHS| values are listed in Supplementary Tables (sheets: GBJ2008-2014_iHS, Basse_iHS).

### Differences in signatures of positive selection between populations

**Figure 2:**
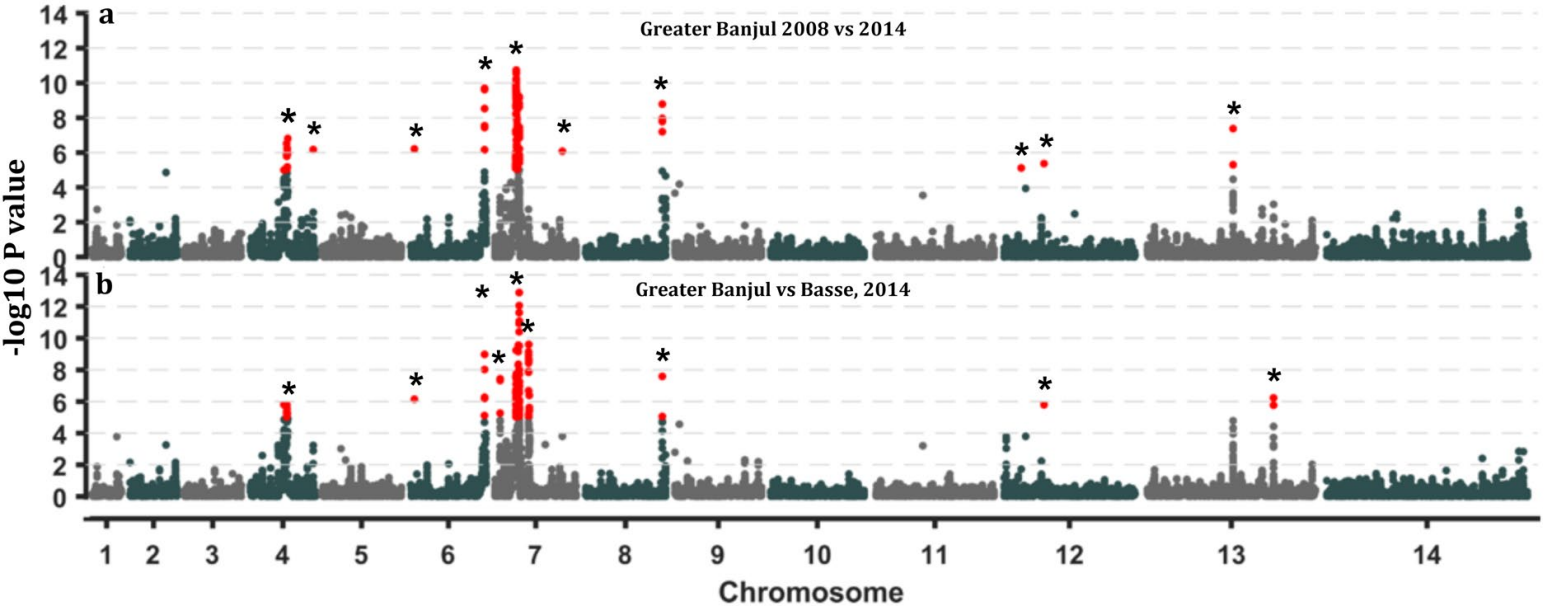
Manhattan plots of −log10 of p-values from the distribution of XP-EHH cross population test for positive selection at genome wide polymorphic coding SNP loci in *P*. *falciparum* populations from The Gambia. (**a**) XP-EHH derived from pairwise comparison of Greater Banjul 2008 against 2014 isolates. (**b**) XP-EHH derived from comparing 2014 population from Greater Banjul with that from Basse. Regions with SNP loci above a set significance threshold (p = <10^−5^) are marked with an asterisk.

Genomic loci with differences in recent selective signatures between populations were consistent for both the relative integrated extended haplotype homozygosity test between populations (Rsb) and the cross population extended haplotype homozygosity (XP-EHH) index (Fig. 2 and Supplementary Fig. 4)^27^. We identified 15 genomic regions (20–220 Kb, >10 SNPs and Wstat score >5) with differences in the range of extended haplotypes between population pairs (Table 1). Five of these regions on chromosomes 4, 6, 7, 8 and 13 were significantly different in both temporal and spatial comparisons of extended haplotypes. The regions on chromosome 4 and 7 spanned the known sweeps in drug resistance associates genes- *Pfdhfr* and *Pfcrt* respectively, while that on chromosome 6 had also been previously characterised^28^. We found relatively reduced lengths and frequencies of extended haplotypes in the 2014 populations for SNPs within the following drug resistance marker codons- *Pfdhfr*-S108N, *Pfcrt*-K76T, *Pfmdr1*-Y184T and *Pfdhps*-S436A (Supplementary Fig. 5). The bifunctional dihydrofolate synthetase–folylpolyglutamate synthetase (*Pfdhfs*-*fpgs*) located on chromosome 13 had significant differences in extended haplotype between the temporal population pair (identified only by Rsb), though it is not targeted by antifolate antimalarial regimes^29^.

**Table 1:** Top windows of significant differences in signatures of positive selection.

*CHR*	*Start* (*kbp*)	*Stop* (*kbp*)	*Size* (*kbp*)	*SNPs* (*n*)	*W*_*temp*_ *Rsb*	*W*_*temp*_ *XP-EHH*	*W*_*spatial*_ *Rsb*	*W*_*spatial*_ *XP-EHH*	Gene IDs within window
*3*	350	390	40	46	6.2	6.3			PF3D7_0307900–0309200
*3*	530	570	40	20		5.3			PF3D7_0312500–0314100
*4*	630	810	180	162	9	17.4	20.3	21.9	PF3D7_0414000–0418000*
*5*	470	690	220	228	5.4	12.3			PF3D7_0511200–0516600
*6*	1230	1270	20	15	10.3	12.5	19.2	12.4	PF3D7_0629700–0630300*
*7*	350	530	180	193	7	13.9	49.4	68.3	PF3D7_0707500–0712000*
*7*	570	690	120	117	13		23	23.3	PF3D7_0712600–0715500*
*8*	1350	1370	20	30	8.2	11.7		5.5	PF3D7_0831500–0831700*
*9*	210	290	80	69	7.6	5.7			PF3D7_0904500–0905800
*10*	1430	1490	60	48	5.5				PF3D7_1036300–1037500
*11*	210	290	80	51	5.8	6.5			PF3D7_1104900–1106900
*12*	62	110	47.9	66	8.7				PF3D7_1200700–1202000
*13*	1030	1070	40	17	5.4				PF3D7_1324600–325700
*13*	1450	1530	80	94	8.1		6	5.4	PF3D7_1335400–1337800*
*13*	2110	2130	20	29	11.4				PF3D7_1352700–1353100

Candidate regions were defined when at least 10 SNPs within a 20kbp window had significant differences in extended haplotype homozygosity measured by cross population indices Rsb and XP-EHH between the temporal population pair from Greater Banjul (2008 versus 2014) and the spatial pair (2014 Greater Banjul and Basse populations). Physical windows (kbp)with a Wstat threshold from GenWin spline analysis between temporal and spatial pairs are represented as W_temp_ and W_spatial_ respectively for each index. Rows with common windows between temporal and spatial pair comparisons are marked with * in the last column. The first column presents the chromosome number (CHR).

Evidence of recent positive selection on antigenic loci was also supported by Rsb and XP-EHH analyses. The signal on chromosome 8 spanned three export proteins and the invasion associated gene, *Pfclag8*, while that on chromosome 13 was around the *Pfmsp7*-like cluster and *Pftrap*. SNPs in the *Pftrap* region had the highest |iHS| in the Basse population, indicating continuous selection on these antigenic loci. Other antigenic regions of differential positive selection were coding for *Pfdblmsp2* and *Pflsa* on chromosome 10; *Pfresa* and *Pflsap1* on chromosome 12, which were specifically different by Rsb between temporal population pairs (Table 1). All candidate genes within positive selection windows by Rsb and XP-EHH are listed in Supplementary Tables (sheet: Rsb_XPEHH_Top Genes).

### A chromosome 5 signature of positive selection in the recent GBJ-2014 population

The widest genomic region with clusters of SNPs with extended haplotype differences between the pair of temporal populations spanned 220 Kb on chromosome 5. SNPs with the highest Rsb value in this region were within the coding frame of tRNA pseudouridine synthase, putative (PF3D7_0516300), downstream of the cation transporter *Pfatpase1*. Examination of the decay of haplotypes in this region showed increased frequencies of haplotypes on the non-reference allele in the current GBJ 2014 population (Supplementary Fig. 6). The region codes mostly for metabolic proteins (PlasmoDB). Three of these are involved in inositol pathways, including phosphatidylinositol 3-kinase (*Pfpi3k*, PF3D7_0515300), which is linked to artemisinin resistance mechanisms^30^.

### Allele frequency differences between temporal population pair

**Figure 3:**
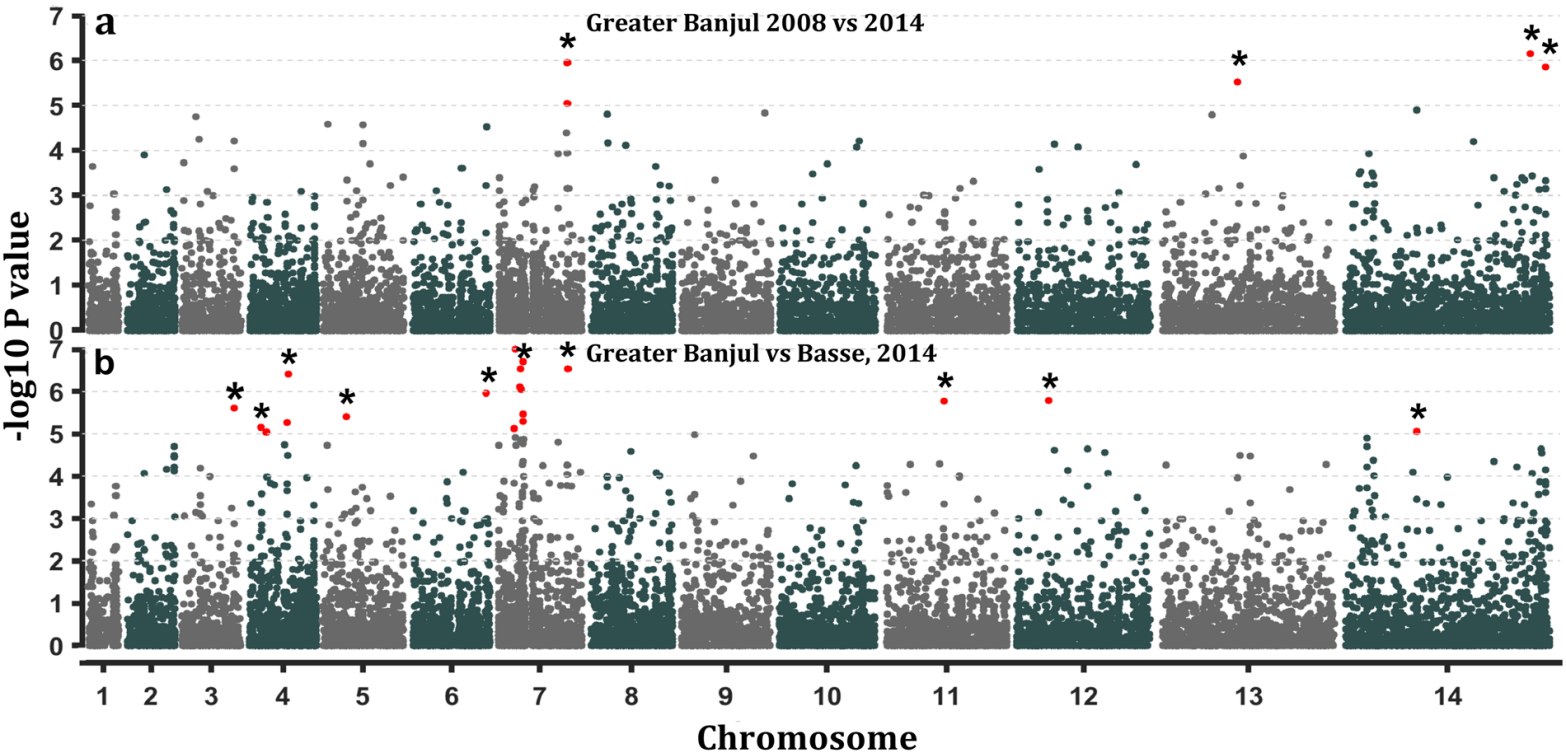
Manhattan plots the −log10 of p-values for pairwise index of fixation *F*_ST_ per coding SNP between temporal (Greater Banjul 2008 versus 2014) and spatial (Greater Banjul 2014 versus Basse in 2014) *P*. *falciparum* population pairs from The Gambia; (**a**) Weir and Cockerham’s *F*_ST_ between temporal populations and (**b**) *F*_ST_ between spatial populations. SNP loci with indices above the threshold of significance (p = <10^−5^) are marked with an asterisk.

We found 6 SNPs significantly (−log10Pvalue ≥ 5) differentiating by population fixation index (*F*_ST_) between our temporal population pair on chromosomes 7, 13 and 14 (Fig. 3a). The strongest evidence of temporal differentiation was observed for a non-synonymous SNP (ns-SNP) on a metabolic gene, cysteine desulfurase (*Pfnfs*, PF3D7_0727200), which had three ns-SNPs with high *F*_*S*T_ values. Sliding window analysis of *F*_ST_ values located a 27kbp haplotype around *Pfnfs* on chromosome 7 with significant differences in frequencies between the temporal population pair (Table 2 and Supplementary Fig. 6). SNPs in two genes upstream *Pfnfs*; mitochondrial import inner membrane translocase subunit TIM50, putative (*Pftim50*, PF3D7_0726900) and vacuolar protein sorting-associated protein 53, putative (*Pfvpsa53*, PF3D7_0727000) were also identified as outliers by the extended Lewontin and Krakauer (LK) test, FLK, between temporal populations (Supplementary Fig. 7a). The widest region with a cluster of differentiating SNPs between these populations was the 220 kbp selective signature identified by Rsb on chromosome 5 (Table 2). We further showed by hapFLK that haplotype frequencies around tRNA pseudouridine synthase within this chromosome 5 region differ between our temporal population pair from GBJ (Supplementary Fig. 7b). Differences in haplotype frequencies on the tRNA pseudouridine synthase on chromosome 5 and cysteine desulfarase in chromosome 7 were more significant between temporal population pair from the GBJ region. Other top hapFLK outliers included *Pfclag2* on chromosome 2 and an exported protein *Pfphistb* next to *Pfsurfin1*4.*1* on chromosome 14. These results confirm differences in the sweep signatures detected in these regions by Rsb and XP-EHH between the temporal population pair. The chromosome 5 region is ~200 kbp upstream of the known *Pfmdr1* sweep on chromosome 5, which was not detected by our genome scan.

**Table 2:** Genomic windows of significant differentiation between temporal (Greater Banjul 2008 versus 2014) and spatial (Greater Banjul against Basse from 2014) *P*. *falciparum* population pairs.

*CHR*	*Start* (*kbp*)	*Stop* (*kbp*)	*Size* (*kbp*)	*SNPs* (*n*)	*W*_*temp*_ *F*_*ST*_	*W*_*spatial*_ *F*_*ST*_	Gene IDs within window
*2*	828.7	860.9	32.2	94	4.7	11.34	PF3D7_0220600–PF3D7_0221400
*4*	630.1	762.3	132.2	32	4.53	5.75	PF3D7_0414000–PF3D7_0417400
*5*	637.9	786.9	149	94	5.18	1.92	PF3D7_0515400–PF3D7_0518900
*6*	1207.9	1242.1	34.2	18	5	3.17	PF3D7_0629400–PF3D7_0629700
*7*	388.1	437.9	49.8	74	2.61	5	PF3D7_0708500–PF3D7_0709800
*7*	1140.3	1167.3	27	35	7.27	1.23	PF3D7_0726600–PF3D7_0727500
*11*	938.5	1058.7	120.2	78	1.45	5	PF3D7_1123700–PF3D7_1127000
*13*	238.3	380.7	142.4	109	6.24	0.25	PF3D7_1304600–PF3D7_1308400

Windows, in kilobase pairs (kbp), with more than 10 SNPs and Wstat threshold of at least 5 (bolded fonts) from GenWin spline analysis of *F*_ST_ values were considered significant and shown as W_temp_ and W_spatial_ for temporal and spatial *F*_ST_ values respectively. The first column presents the chromosome number (CHR).

### Allele frequency differences between spatial population pair

In line with the higher genome-wide fixation index between the spatial population pair, more SNPs (20) were significantly differentiating between GBJ and Basse isolates collected in 2014 (Fig. 3b). Half of these were located on chromosome 7, spanning the region coding for the chloroquine resistance gene (*Pfcrt*), (Supplementary Fig. 7c). However, the strongest indication of spatial differentiation by *F*_ST_ was on a s-SNP in the antigenic gene, rhoptry-associated membrane antigen (*Pframa*, PF3D7_0707300). High *F*_ST_ at ns-SNPs clustered within the coding frame of a conserved protein of unknown function (PF3D7_0710200), which has homologous domains with reticulocyte binding protein 1 (*Pfrh1*). This gene had significant *F*_ST_ values for 5 ns-SNPs and 1 s-SNP. The widest window of SNPs with high *F*_ST_ values between the spatial population pair was on chromosome 4, spanning the drug resistance locus *Pfdhfr*, which also showed sweep differences detected by Rsb and XP-EHH between the spatial population pair. SNPs around *Pfdhfr* were within 0.01% of top hits for haplotype frequency differences between spatial populations determined by spatial hapFLK (Supplementary Fig. 7d). Differential selection on haplotypes in the autophagy-related relate protein (*Pfatg7*, PF3D7_1126100) on chromosome 11 was specifically detected by hapFLK between spatial population pair. Evidence of differentiation on haplotypes common to both temporal and spatial comparisons population pairs were seen on *Pfclag2* on chromosome 2, *Pfset1* on chromosome 6 and *Pfsurfin14*.*1* on chromosome 14. Indices of differentiation for SNPs are listed in Supplementary Tables file (sheets: FLK_Temporal, FLK_Spatial hapflk_temporal, hapflk_Spatial).

### Directional selection of *Plasmodium falciparum* cysteine desulfarase variant in GBJ populations

**Figure 4:**
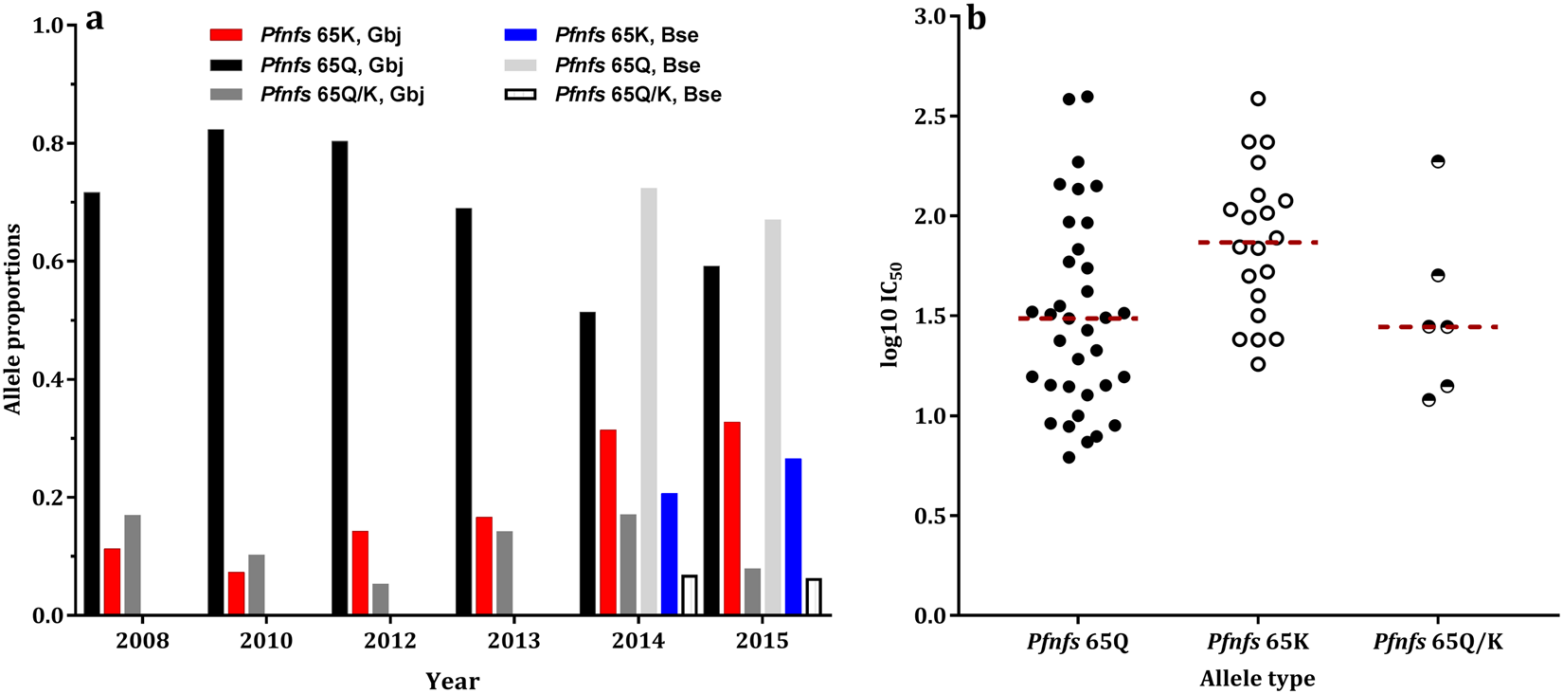
Prevalence of variant alleles at position 1,158,217 of chromosome 7, in the cysteine desulfarase (NFS; Pf3D7_0727200) in clinical *Plasmodium falciparum* isolates from the Gambia; (**a**) the proportions of alleles in isolates from clinical malaria samples collected from Greater Banjul between 2008 and 2015. (**b**) Scatter plot of the lumefantrine IC_50_ concentrations for isolates with *Pfnfs*-*65K* (reference type) or *Pfnfs*-*65Q* (non-reference) allele.

We determined changes in frequencies of alleles at Pf3D7_07_v3:1158217 within cysteine desulfarase (*Pfnfs*) gene in 521 *P*. *falciparum* isolates collected yearly between 2008 and 2015. The reference allele at this locus codes for the polar charged amino acid, lysine (K) at codon 65 of *Pfnfs*. The alternative allele codes for the polar but uncharged amino acid, glutamine (Q). Allelic discrimination analysis of temporally collected isolates spanning this period from GBJ showed a steady increase in the frequency of the reference type allele of the *Pfnfs* SNP from 2010 (Fig. 4a)_._ The *Pfnfs*-*6*5*K* reference allele was present in 10% of clinical malaria samples from the GBJ region, at the time of introduction of ACTs in 2008 in the country. Its frequency dropped slightly to 7% by 2010 and then rose steadily to 32% of isolates by 2015. About 5–10% of isolates from mixed infections had both alleles. To explore if alleles at the *Pfnfs* locus have any association with drug responses, we analysed their distribution in isolates tested *in vitro* for susceptibility to lumefantrine in 2015^23^. We found significantly higher IC_50_ concentrations for lumefantrine in *P*. *falciparum* isolates from GBJ with the reference lysine variant at *Pfnfs* locus (p = 0.0029), (Fig. 4b). We found lower frequencies of this variant in clinical isolates collected in 2014 and 2015 from the higher transmission region of Basse. However, the reference allele frequency also increased by 6% (from 20% to 26%) within one year in the Basse population. Allele frequencies at SNP-1,115,521 on chromosome 8 within HECT-like E3 ubiquitin ligase, putative (PF3D7_0826100) were similar for the 2008 and 2015 GBJ populations.

## Discussion

*Plasmodium falciparum* is under intense pressure from malaria control interventions across Africa and this could result in adaptation in some populations^31^. To identify regions of *P*. *falciparum* genome with recent signatures of selection, we compared the genomes of recently collected isolates to those collected in 2008, prior to the introduction of ACTs^32^. We also compared genomes across a gradient of transmission intensity to detect common selective signatures in the more recent populations as well as local signatures that could suggest an effect of variance in intervention, transmission or host characteristics between sites^33,34^. The population pairs analysed showed no significant structure, as expected for African populations. This indicates sustained genetic diversity within The Gambian GBJ population and significant mixing of parasites between GBJ and Basse, despite the 6-years between sampling, intense interventions and spatial separation of 350 km across the country.

Differentiation between the GBJ temporal population pair was most evident at a cysteine desulfarase gene (*Pfnfs*). Cysteine desulfarases are involved in the delivery of sulphur to multiple metabolic pathways, including Fe-S complex biogenesis^35^. Fe-S cluster-containing proteins in a*picomplexans* are modulated during stress conditions, drug resistance and cell development^36,37^. They play vital roles in housekeeping, gene expression and other cellular processes and are targeted by antimalarial drugs^38,39^. Moreover, iron homeostasis is essential for erythrocytic development and is implicated in drug mechanisms of quinolines^40,41^, same drug family like Lumefantrine. We therefore selected the SNP within the *Pfnfs* for an initial analysis of temporal changes in allele frequency. The frequency of the reference and minor lysine variant in *Pfnfs*-codon K65Q increased steadily in the GBJ population, two years after the introduction of ACTs in the country. Low transmission intensity and lower recombination in the GBJ population will favour directional selection of this allele if it confers a selective advantage. Coupled to our recent report of temporal increase in the IC_50_s to Lumefantrine in the GBJ Gambian population^23^, it is plausible that *Pfnfs* may be under selection from Lumefantrine. We believe this is imposed by ACTs as the IC_50_s to Lumefantrine for isolates carrying the lysine allele were higher in the recent GBJ population. Moreover, the increase in the lysine allele frequencies in Basse, further suggest a common driver; such as ACTs, in both populations. Determining if the driver of selection is at linked sites in *Pftim50* and *Pfvps53*, or any of the other 7 genes within the window of differentiation could identify associated markers for Lumefantrine sensitivity monitoring. Tim50 proteins are involved in protein complex formation and trafficking across membranes^42^, while vps53 is a component of the heterotetrameric tethering factor named Golgi-associated retrograde protein (GARP)^43^. We are currently exploring these signatures in other regions of Africa with extensive Artemether-Lumefantrine use.

We found a region of extended haplotype difference between temporal populations on chromosome 5. High *F*_ST,_ FLK and hapFLK indices for SNPs within this window further suggest recent directional positive selection on loci in this region in the GBJ 2014 population. This is the first record of a signature of selection in this region in a recent *P*. *falciparum* population in sSA. The region codes for several metabolic proteins including Phosphatidylinositol 3-kinase, which is associated with artemisinin resistance^30^. The candidate SNPs in the region differentiating between temporal populations were on a tRNA pseudouridine synthase, responsible for the most abundant post-transcriptional modification of cellular RNAs^44^. Further analysis of this candidate region of temporal differentiation for functional association with ACT tolerance is important to determine if the genes coded for are associated with the same mechanisms mediating artemisinin resistance in SEA.

Most of the SNPs differing in frequency between the GBJ and Basse spatial populations where around the drug resistance genes, *Pfcrt* and *Pfdhfr*, suggesting differences in selective pressure from Chloroquine and SP between the sites. However, the strongest differentiating SNPs between the populations were on antigenic loci; *Pframa* by *F*_ST_ and *Pftrap* by differences in extended haplotype. *Pframa* is a GPI-anchored rhoptry protein implicated in merozoite invasion of erythrocytes while *Pftrap* mediates gliding motility and invasion processes of malarial sporozoites into vertebrate’s hepatocyte and mosquito’s salivary gland^45–47^. Selection may be imposed by differences in human population but with increased interventions against the vector, *Pftrap* could also be under selection by the differences in vector populations between coastal GBJ and inland Basse^48^. Though immune selection often results in balancing selection, we identified signatures of recent positive selection on multiple antigenic loci; *Pfclag2*, *Pfama1*, *Pfmsp1*, *Pfdblmsp2*, *Pfresa*, *Pflsa*, *Pfsurfins* and the *Pfmsp7*-like cluster. Signatures of positive selection on antigenic loci have been identified before in *P*. *falciparum* population genome scans^49–51^. Some of these antigenic loci are implicated in invasion mechanisms and new alleles that are occasionally introduced in a population by mutation or immigration would increase in frequency together with nearby variants before being checked by acquired immunity. These signatures were most significant in the Basse population, where exposure and immune selection could be stronger due to the relatively higher malaria transmission. *Plasmodium falciparum* enhances its invasion and immune evasion capacities to meet the environmental stressors, including immunity and drugs within a specific spatial or temporal population. Hence antigen haplotypes enabling fitness in the immune environment would be positively selected. Some of this complex selection pattern could be because of heterogeneity or restriction in recombination rates between allelic variants within a gene^52^.

Selective sweeps around drug resistance loci, *Pfdhfr*, *Pfcrt* and the extensive region on chromosome 6 that coded for *Pfptps* and *Pfmthfd*, were common across all populations analysed. This result is consistent with previous genome scans of West African populations^13,14,32,53^. The extent of extended haplotypes around drug resistance *Pfcrt* and *Pfdhfr* had reduced in recent GBJ population but remained strong in the Basse area with higher transmission. It is anticipated that a decay of selective sweeps would follow the withdrawal of Chloroquine and antifolates as first line treatment for malaria. However, the use of non-ACTs in rural areas such as Basse may be responsible for maintaining selective pressure on *Pfcrt* and *Pfdhfr*. Basse had a lower genome-wide LD than the GBJ population, where LD had increased between the 6 years of sampling. Higher LD in GBJ could be due to increased levels of self-fertilisation as infection prevalence and malaria transmission declined during this period. Higher LD and lower infection complexity has been reported in the Southeast Asian *P*. *falciparum* populations^53,54^, the epicentre of antimalarial resistance development and rapid evolution of selective sweeps. Hence, the increasing LD in GBJ and relatively lower infection complexities could favour sweeps around *de novo* or standing genetic variants associated with drug resistance, threatening the efficacy of antimalarials.

## Conclusion

*Plasmodium falciparum* has a relatively short generation time and stochastic shifts in allele frequencies can occur rapidly as interventions reduce parasite populations. However, allele frequency shifts and extended haplotypes in genes implicated in pathways of adaptation can indicate a process to cope with environmental stress due to interventions. Known genomic signatures of positive selection persist in the Gambian *P*. *falciparum* population and a new extended region has been identified on chromosome 5. We found evidence of directional selection in a *P*. *falciparum* cysteine desulfarase, occurring just 6 years after introducing ACTs. Left unchecked, this could compromise Lumefantrine efficacy and ultimately, AL treatment failure. This is important and underlines the need for continuous surveillance of the role of emerging genetic variants in decreasing ACT efficacy in Africa. The association between *Pfnfs*-6*5k* and Lumefantrine tolerance needs further evaluation in parasite populations from other endemic regions that rely on AL, to guide antimalarial chemotherapy policies.

## Methods

### Study approval

The study was reviewed and approved by MRCG scientific coordinating committee. Ethical approval for sample collection and for the use of the retrospective genomic data and samples from 2008–2011 was obtained from the Gambian Government-MRCG Joint Ethics Committee. A signed informed consent was obtained for all participants above 18 years or from parents or guardians for those below 18 years. All sample and data handling methods were performed in accordance with the relevant guidelines and regulations at MRCG.

### Parasite isolates

Between 2012 and 2015, patients diagnosed with uncomplicated malaria at Brikama and Basse health centres were invited to participate in the study. After obtaining a written informed consent, a 2 ml venous blood sample was collected from each participant. Brikama is a town in the Greater Banjul (GBJ) region on the western end, while Basse is about 350 km away on the eastern end of country. These sites reflect the gradient of transmission across the country, with low transmission in the west (5–15% parasite prevalence) and moderate to high transmission in the east (15–50% parasite prevalence)^20^. *Plasmodium falciparum* isolates had been collected from western Gambia between 2008 and 2011 at the Royal Victoria Teaching Hospital in Banjul and the Brikama Health centre and stored at the Medical Research Council Unit The Gambia (MRCG) biobank. Isolates from 2008 had been sequenced and analysed previously for signatures of selection^32^.

### Genome sequencing

*Plasmodium falciparum* parasite genomic DNA for sequencing was extracted from whole blood depleted of leukocytes by filtration through CF11 cellulose columns as previously described^55^. Sequencing was done on Illumina HiSeq platform in collaboration with the MalariaGEN consortium at the Wellcome Trust Sanger Institute, Hinxton, UK^56^. Paired end DNA sequence-reads (150 bp) were obtained from 44 and 124 isolates collected in 2014 from western and eastern Gambia, respectively. Together with reads from 79 isolates sequenced in 2008, they were aligned to the *P*. *falciparum* 3D7 reference genome (version 3) as previously described^6,57^. Nucleotide variants were called using custom in-house calling algorithms, filtering and retaining the dominant allele at bi-allelic coding variable sites in a genotype file. Each population was genotyped for 791,385 high quality SNPs supported by a minimum of five short sequence reads as defined by MalariaGEN/Pf3K pipeline. Binary haplotype data was created denoting variable nucleotides as reference (0) or non-reference (1) allele. The short reads are available via the ENA database (www.ena.org).

### Population complexity, structure and linkage disequilibrium

For each isolate, infection complexity was determined by Fws as previously described^57^. Pairwise SNP linkage disequilibrium (LD) expressed as the coefficient of correlation (r^2^) between alleles at physically separated loci with a minor allele frequency (maf) of at least 2% across all populations was calculated in plink. A LOESS spline was fitted for r^2^ between loci within 100 kbp to compare the decay in LD with physical distance in each population. To determine if there was structure between the temporal and spatial populations, all SNPs at an LD threshold (r^2^) < 0.5 were used to generate a pairwise (nxn) matrix of shared alleles between isolates, with n as isolates. The matrix was employed in principal component analysis, with the first and second principal components for each isolate plotted for 2008 against 2014 GBJ populations, and for GBJ against the Basse 2014 populations.

### Signatures of selection

To detect loci under recent positive selection, we calculated the extended haplotype homozygosity (EHH)–derived indices; standardised intra-population integrated haplotype score (|iHS|), and the cross-population ratio of the EHH expressed as Rsb and XP-EHH for each SNP with the R package REHH^58^. For iHS analysis, the reference and non-reference alleles were employed in lieu of the ancestral and derived alleles, respectively. For significance, the REHH package generates a two-sided p-value as

$$-\,\mathrm{log}\,10(1-2|{\rm{\Phi }}({\rm{iHS}})-0.5|),$$

where Ф(iHS) represents the Gaussian cumulative distribution function. For selected significant core markers, the decay in haplotype frequencies in each population was examined with decay and bifurcation plots in R.

### Population differentiation

Differentiation at SNP loci between population pairs were analysed using Wier and Cockerham’s *F*_ST_ per SNP, calculated with the *hierfstat* package in R. The variance component of *F*_ST_ between the pair of temporal and spatial populations were also determined in *hierfstat*. The significance of *F*_ST_ values were determined by sampling from ten million permutations of the *F*_ST_ distribution for each pairwise comparison. We minimised the false positive rate for differentiating loci by computing the extended Lewontin and Krakauer test, FLK, and the linked derivative hapFLK, which focuses instead on the differences of haplotype frequencies between populations^59,60^. The FLK index accounts for population size heterogeneity and possible hierarchical structure between populations. It computes a global *F*_ST_ for each SNP, but allele frequencies are first rescaled using a population kinship matrix, estimated from the observed genome wide data. With this rescaling, allele frequency differences obtained with small populations are typically down-weighted. HapFLK is an extension of FLK, integrating a multipoint linkage disequilibrium that regroups individual chromosomes into local haplotype clusters before measuring differentiation between populations^59^. A software for calculating FLK and HapFLK is free available on from (https://forge-dga.jouy.inra.fr/projects/hapflk). A p-value of 10^−5^ for *F*_ST_ and FLK was arbitrarily set as threshold for significance.

### Outlier genomic windows of selection and differentiation

To determine genomic windows with contiguous significant signatures, we fitted a spline on *F*_ST_, Rsb and XP-EHH values per chromosome with the GenWin package in R using genome-wide mean and variance values to normalise across chromosomes. GenWin defines a t-test like statistic, W, generated such that:

$$W=\frac{x-\mu }{\sqrt{({s}^{2}/n)}}$$

where x is the mean value over the window, μ is the mean value over the entire dataset, s^2^ is the sample variance of index across the genome, and n is the number of observations (i.e. markers) in the window^61^. Windows of 5000 to 25000 bp were assessed and overlapping segments with a W value > =5, with more than 10 SNPs were considered significant. Functional annotations for genes within significant windows were obtained from a combination of tools defining gene ontologies in PlasmoDB, PANTHER, DAVID, LAMP and gene ontology tools (http://geneontology.org/page/go-enrichment-analysis).

### Temporal changes in allele frequency

The non-synonymous SNP at physical position 1,158,217 of chromosome 7 within the *P*. *falciparum* cysteine desulfarase gene (PF3D7_0727200) had the most significant temporal difference in allele frequency. We designed Taqman allelic discrimination assay for this polymorphism using the online assay design tool provided by life technologies (Life Technologies assay ID: AN7DRNA). We also designed an assay for a randomly chosen SNP at position 1,115,521 on chromosome 8 within HECT-like E3 ubiquitin ligase, putative (PF3D7_0826100), (Life Technologies assay ID: ANRWF4A). We genotyped these loci in 521 *P*. *falciparum* isolates from GBJ region collected in 2008 (53), 2010 (68), 2012 (57), 2013 (42), 2014 (35) and 2015 (125); and from Basse (eastern region) in 2014 (60) and 2015 (81). Furthermore, we determined the difference in Lumefantrine IC_50_ values for isolates with the reference or non-reference allele for 61 isolates assayed from the 2015 GBJ population. Mann Whitney t-test was used to determine the significance of the difference in mean IC_50_s between allele-groups with a significance level set at 0.05.

### Data availability

Short sequence reads analysed are available via European Nucleotide Archive.

## Electronic supplementary material

Supplementary figures

Supplementary Dataset 1
