# Supplementary material for: Consistent signatures of selection from genomic analysis of pairs of temporal and spatial Plasmodium falciparum populations from The Gambia
Source: Sci Rep. 2018 Jun 26;8:9687. doi: 10.1038/s41598-018-28017-5 (PMC6018809; doi:10.1038/s41598-018-28017-5)

**Supplementary Figures**

**Title: Consistent signatures of selection from genomic analysis of pairs of temporal and spatial Plasmodium falciparum populations from The Gambia**

Alfred Amambua-Ngwa^1^*, David Jeffries^1^, Roberto Amato^2^, Archibald Worwui^1^, Mane Karim^1^, Sukai Ceesay^1^, Haddy Nyang^1^, Davis Nwakanma^1^, Joseph Okebe^1^, Dominic Kwiatkowski^3^, David J. Conway^2^, Umberto D’Alessandro^1,2^

1 Medical Research Council Unit The Gambia at LSHTM, 2 London School of Hygiene and Tropical Medicine (LSHTM), London, UK, 3 Wellcome Trust Sanger Institute, Hinxton, UK

*Corresponding author (angwa@mrc.gm)

Supplementary Figure 1: Spline smoothing of linkage disequilibrium (LD) measured as r^2^ (y-axis) against physical distance (bps) between SNPs for each chromosome labelled 1 to 14 (x-axis). The strongest LD is between SNPs for the more recent 2014 population from Greater Banjul, while the least is for SNPs from the Basse population collected in 2014. Long range LD were detected on all chromosomes from the population from Greater Banjul (2008 and 2014).


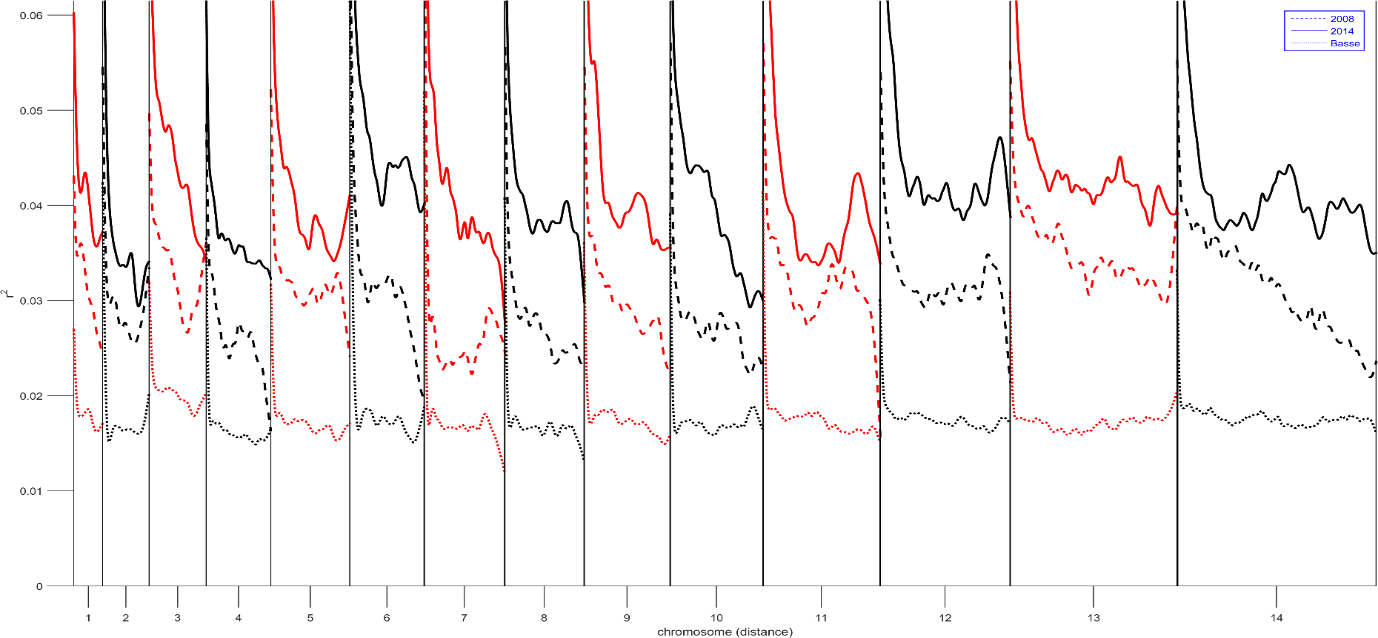


Supplementary Figure 2: Population structure of three *P. falciparum* populations collected from Greater Banjul in the West of the Gambia in 2008 and 2014, and from Basse in the East in 2014. Principal component projections PC1 is plotted against PC2 for each population pair. (a) Greater Banjul 2008 (red) and 2014 (black), (b) Greater Banjul 2014 (red) and Basse 2014 (black).


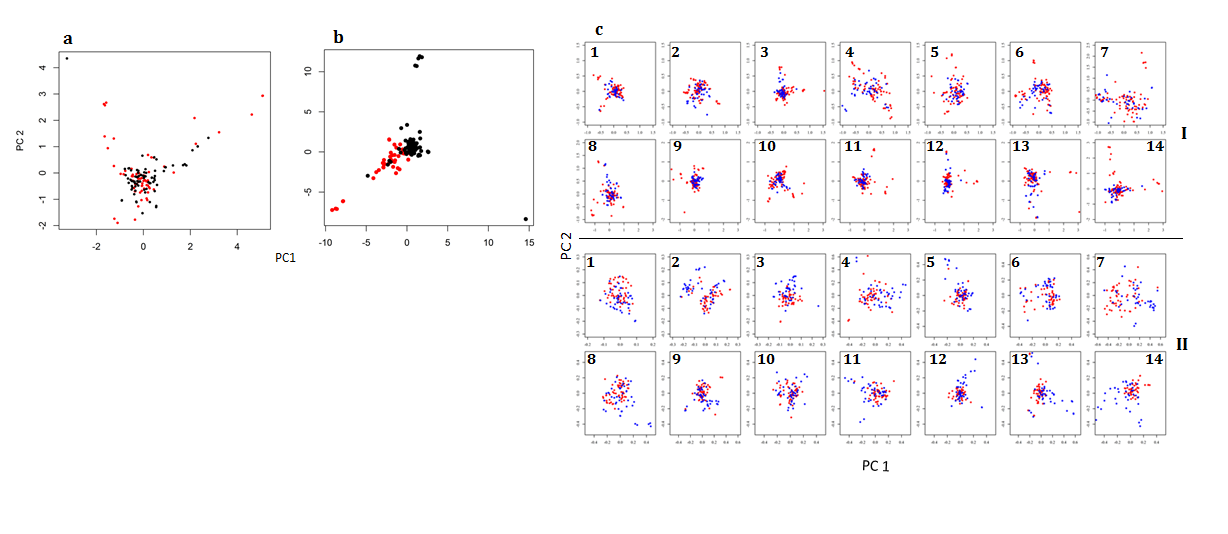


Supplementary Figure 3: Manhattan plots of signatures of selection from extended haplotype homozygosity test |iHS|. Each point is –log10 p-value of |iHS| for coding SNPs in a) Greater Banjul 2014 b) Greater Banjul 2008 and c) Basse in 2014 *P. falciparum* populations from the Gambia. SNP loci with indices above the threshold of significance (p</=10^-5^) were considered as outliers.
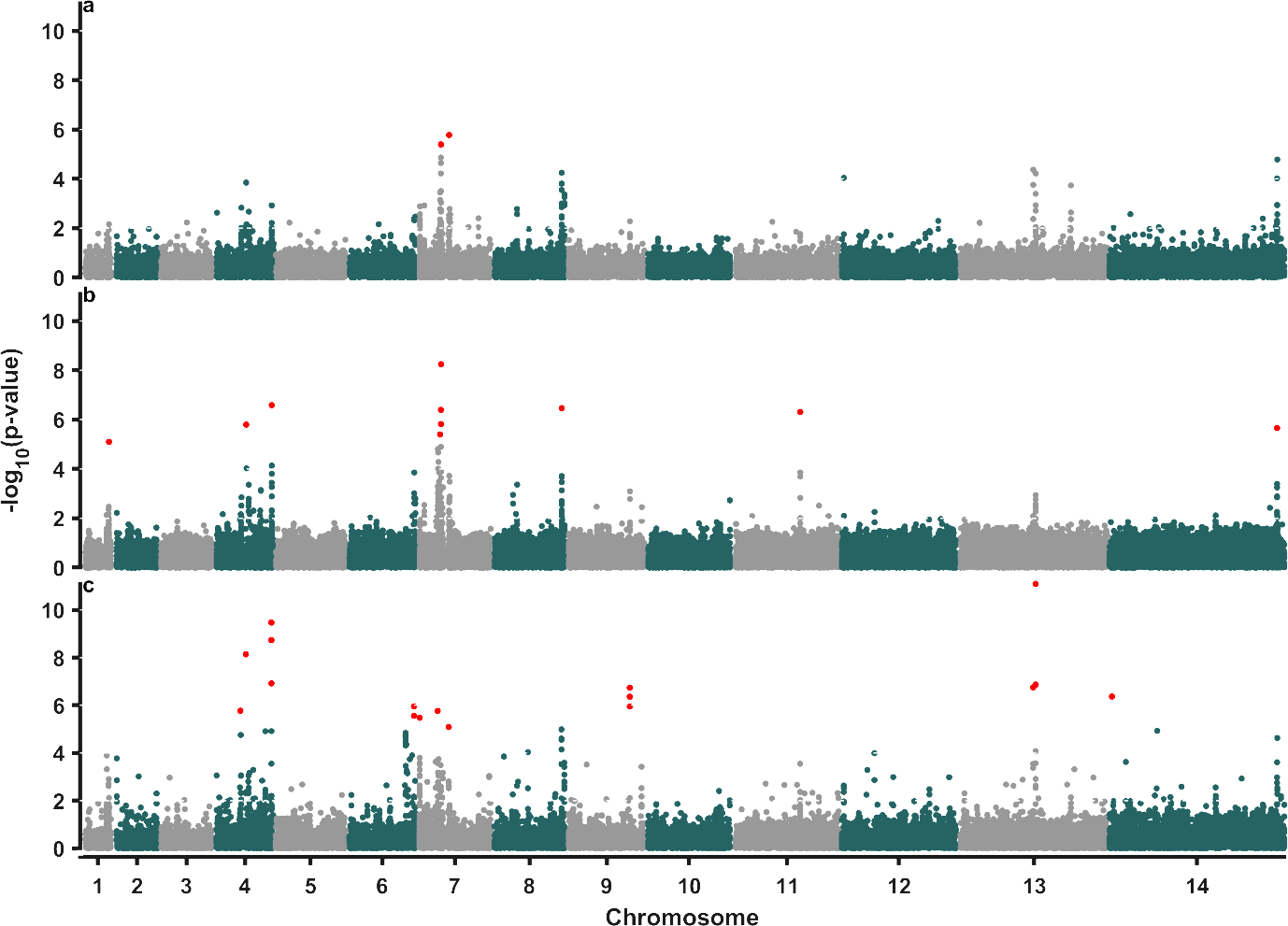


Supplementary Figure 4: Manhattan plots of the –log10 of p-values for pairwise extended haplotype difference determined by Rsb per coding SNP between a) Greater Banjul 2008 and 2014 and, b) Greater Banjul 2014 and Basse in 2014. SNP loci with Rsb p-value<=10^-5^ were considered as under positive directional selection on a haplotype in one population.
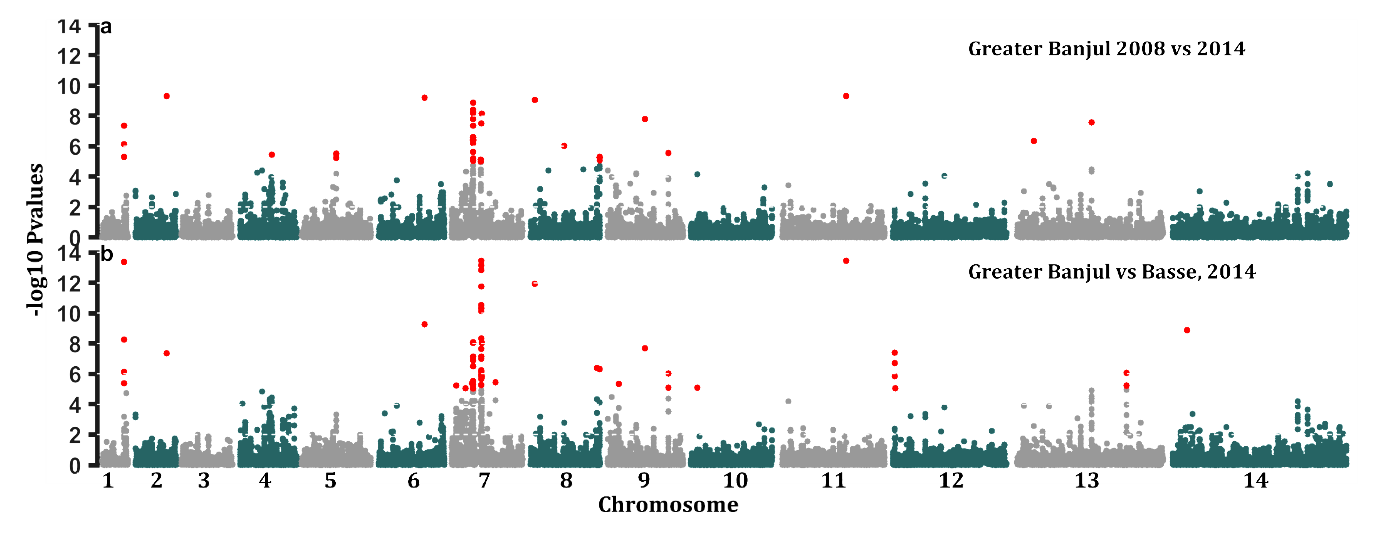


Supplementary Figure 5: Haplotype bifurcation plots at known drug resistance associated SNPs. Panels from top to bottom show (i) Pfdhfr-S108N, ii) Pfmdr-Y184F, iii) Pfcrt-K76T and iv) Pfdhps S436A. Plots were derived from extended haplotype homozygosity (EHH) for SNPs of isolates from the Greater Banjul obtained in 2008 (left column) and 2014 (right column). Vertical lines on each plot represents the physical position of the focal SNP. Physical positions of extended haplotype on chromosomes are denoted by the horizontal axis.


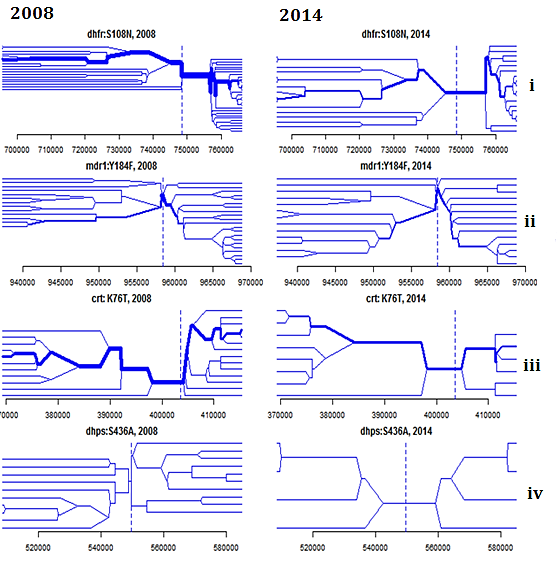


Supplementary Figure 6: Regions of positive selection and differentiation on chromosome 5 (left column) and chromosome 7 (right column) between temporal population pair from Greater Banjul. The first row presents the –log10 p-values for *F*_ST_ for SNPs in chromosomes 5 and 7. Horizontal lines demacate 100kbp region of high *F*_ST_ SNPs. The second row presents a zoom on the focal SNP with the highest *F*_ST_ value, showing extended haplotype decay in the recent 2014 population from Greater Banjul for each of the chromosomal regions. The third row presents extended haplotype decay in the 2008 population from Greater Banjul for each of the focal chromosomal regions. The bottom row is a snapshot of the regions with a candidate signature of selection in the 2014 Greater Banjul population, presenting annotated coding sequences as red or blue bars directed in the sense of the coding strand on *P. falciparum* genome version 3, PlasmoDB.


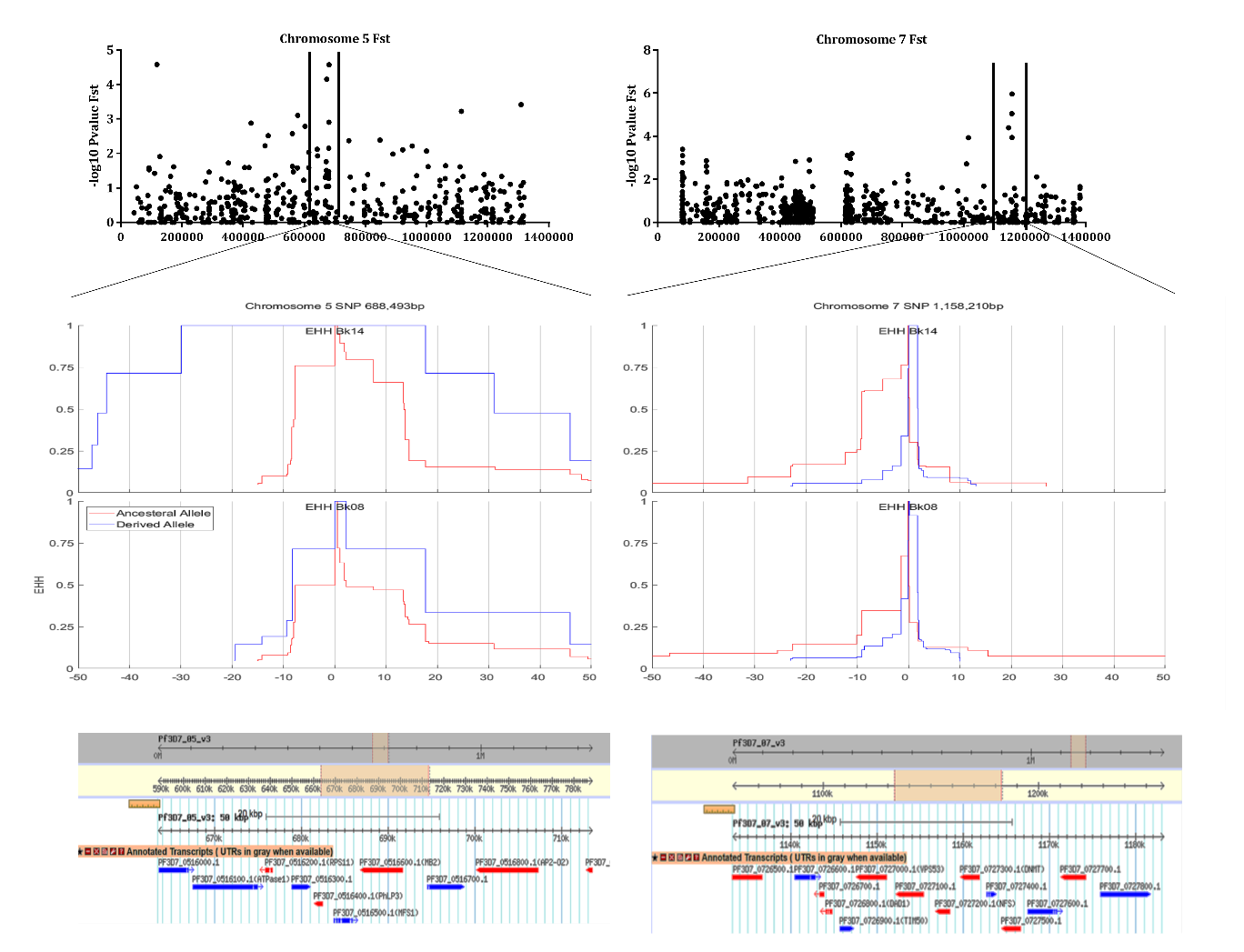


Supplementary Figure 7: Differentiation at SNP loci between the pair of spatial and temporal populations from the Gambia. Each point on the Manhattan plots represent the –log10 of the p-value for a) FLK between isolates from Greater Banjul from 2008 against 2014 with Basse 2014 as an out-group b) hapFLK between isolates from Greater Banjul from 2008 against 2014 with Basse 2014 as an out-group c) FLK between isolates from Greater Banjul from 2014 against Basse 2014 with Greater Banjul 2008 as an out-group, and d) hapFLK between isolates from Greater Banjul from 2014 against Basse 2014 with Greater Banjul 2008 as an out-group.


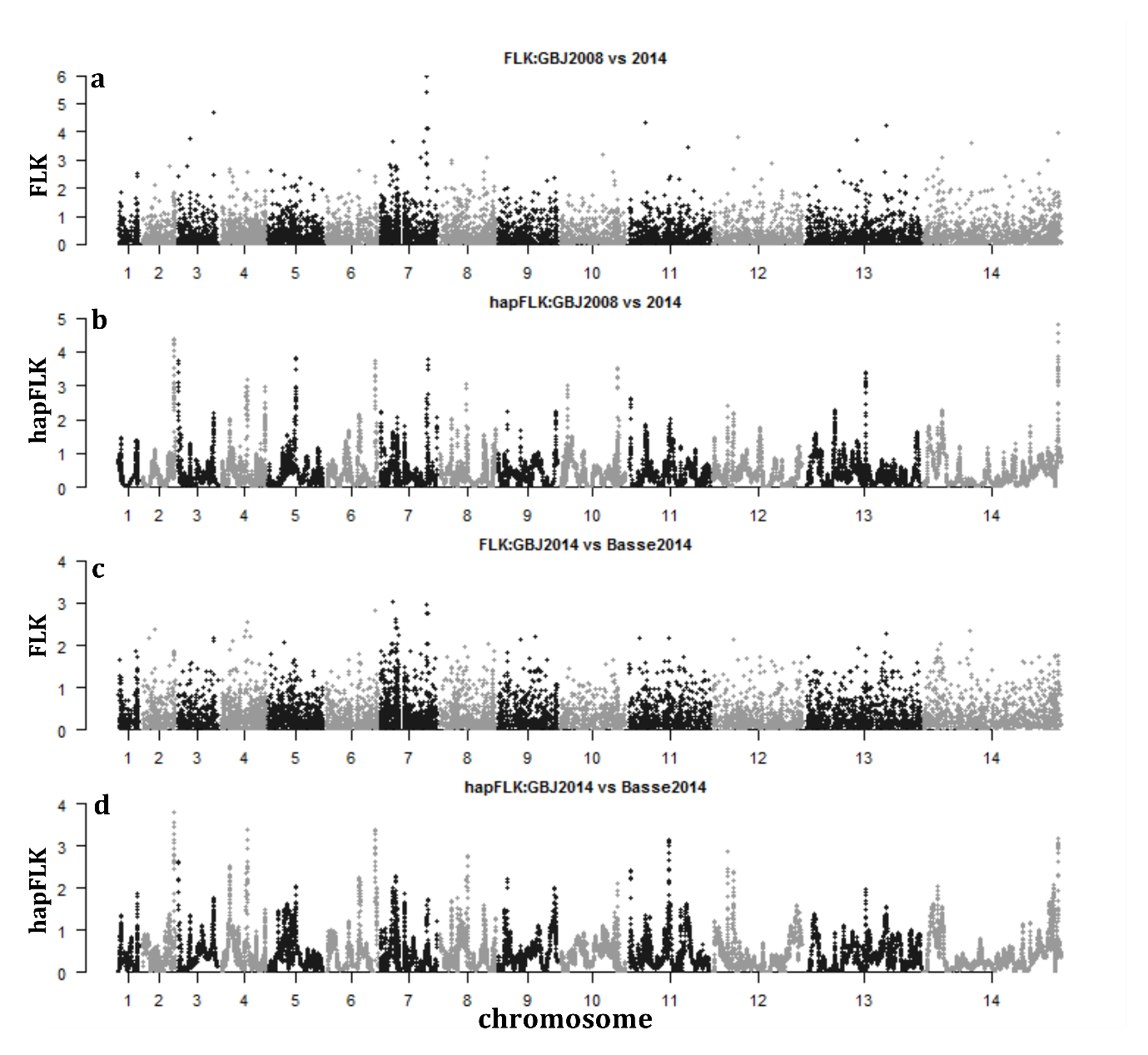

Supplement: Supplementary file 1 — Supplementary figures [file 41598_2018_28017_MOESM1_ESM.doc]
